# Video Ethogram of Equine Social Behaviour

**DOI:** 10.3390/ani14081179

**Published:** 2024-04-14

**Authors:** Laura Torres Borda, Zsofia Kelemen, Ulrike Auer, Florien Jenner

**Affiliations:** 1 Equine Surgery Unit, Department of Companion Animals and Horses, University of Veterinary Medicine Vienna, Veterinaerplatz 1, 1210 Vienna, Austria; laura.torres-borda@vetmeduni.ac.at (L.T.B.); zsofia.kelemen@vetmeduni.ac.at (Z.K.); 2 Anaesthesiology and Perioperative Intensive Care Medicine Unit, Department of Companion Animals and Horses, University of Veterinary Medicine, 1210 Vienna, Austria

**Keywords:** horse, equine, ethogram, social behaviour, sociality, welfare, quality of life

## Abstract

**Simple Summary:**

Comparisons across equine social behaviour studies are currently impeded by the lack of a universally accepted ethogram. Therefore, this ethogram introduces standardised definitions for thirty-seven distinct equine social behaviours, drawing from existing ethograms and refining definitions through meticulous video observations. The definitions consider contextual cues, such as ear position and facial expressions, and communicative nuances. Video examples enhance clarity by capturing the dynamic flow and sequence of social interactions. This approach allows researchers to observe temporal aspects like sequence, duration, and rhythm, providing a detailed representation of equine social behaviours. Standardized definitions, along with video illustrations, promote clear communication among researchers and enable consistent cross-study comparisons, which, in turn, can contribute to a better understanding of how husbandry practices and health conditions impact equine social behaviour, aiding in the assessment and optimization of management practices to enhance equine welfare.

**Abstract:**

Equine social behaviour studies face challenges stemming from the absence of a comprehensive ethogram with unequivocal standardised definitions and the resulting limits to data comparison across studies. To address these constraints, this ethogram offers researchers a standardised framework, defining thirty-seven distinct equine social behaviours supplemented by video examples for enhanced clarity. These definitions amalgamate insights from existing ethograms and are fine-tuned through meticulous video observations, encompassing contextual cues such as distinguishing between aggressive and playful circling based on ear position and facial expressions and communicative nuances to provide a detailed representation of equine social behaviours. Video recordings complement the standardised definitions by capturing the dynamic flow and sequence of social interactions. By providing a dynamic and detailed representation, videos allow researchers to observe the temporal aspects of behaviour, including the sequence, duration, and rhythm of interactions. These detailed data are crucial for interpreting social behaviours and unravelling the complexities of equine societies. Standardized and video-illustrated definitions of equine social behaviour facilitate clear and consistent communication between researchers, enabling cross-study comparisons regarding the impact of husbandry practices and health conditions on equine social behaviour, which, in turn, can facilitate the assessment and optimisation of management practices and equine welfare.

## 1. Introduction

Horses are highly social animals that, under naturalistic conditions, live in stable social groups, characterised by enduring bonds and established dyadic interaction patterns. Their sophisticated repertoire of communicative behaviours and intricate social cognition enables them to navigate their complex social structures, maintain long-term affiliative bonds, and resolve conflicts effectively [1,2,3,4,5,6,7,8,9,10,11,12,13,14,15,16,17]. Horses demonstrate cross-modal individual recognition, utilising a combination of visual, auditory, and olfactory cues to identify specific group members, even after extended periods of separation [18,19,20,21,22,23,24]. Their ability for long-term memorisation of past interactions allows them to gauge their relative social standing and anticipate the outcomes of encounters with familiar individuals, adjusting their behavioural responses accordingly [18,19,20,21,22,23,24,25,26,27,28,29]. This capacity extends to the transitive inference of social relationships through observation [18,26,28]. 

Horses exhibit preferences for specific group members, with whom they form enduring social bonds that are established and maintained by affiliative behaviours [9,13,16,17,30,31,32,33,34]. Intriguingly, horses exhibit third-party interventions in both agonistic and affiliative dyadic interactions among group members and an increase in affiliative behaviours following a conflict, suggesting a sophisticated understanding of social dynamics and an ability to manage social tension [26,28,34]. Correspondingly, within stable (no change in group composition for >2–3 months [35]) horse groups, agonistic behaviours, though present, are infrequent and often ritualised [35,36,37]. However, to date, most studies of equine social behaviour focus on agonistic interactions [7,8,13,26,27,28,34,35,36,37,38,39,40,41,42,43,44]. Indeed, a recent review of equine social ethograms, which included 27 articles [7,8,9,13,17,26,27,28,30,34,35,38,39,40,41,42,43,44,45,46,47,48,49,50,51,52,53], highlighted a dominance of agonistic behaviours, constituting 60% of the 40 non-redundant social behaviours documented across various ethograms [54]. In contrast, affiliative behaviours accounted for only 30%, while investigative behaviours represented 7.5%, with a mere 2.5% allocated to neutral behaviours. The significant roles of affiliative interactions for equine welfare and quality of life, thus, require further studies encompassing the entire repertoire of social interactions [13,35,49].

Comparative studies between feral and domesticated horses have revealed remarkable consistency in equine social behaviour [7]. While quantitative differences were evident with Przewalski horses displaying higher frequencies of social grooming, kick threats and kicks but engaging in less investigative behaviour compared to domesticated horses, the qualitative nature of social behaviours remained alike [7]. However, contrary to their gregarious tendency in naturalistic conditions, most domestic horses are confined to individual stables with limited contact with conspecifics [7,8,13,35,39,48,55,56,57,58,59,60,61,62]. Moreover, their lack of control over group affiliations, frequent changes in social companionship under human management, and potential crowding lead to increased stress, aggression, and frequency of agonistic encounters and corresponding concerns regarding equine welfare and quality of life [8,34,35,39,58,63,64,65,66,67,68,69,70,71,72,73,74,75].

Current equine social ethograms predominantly derive from observations of equine bachelor groups and include non-standardized descriptions of variable subsets of social behaviour complemented solely by drawings or photos with the inherent limitations of static representations of a dynamic process [30,38,44,75,76,77,78]. Consequently, comparison between studies is challenging, thus restricting the comprehensive assessment of the impact of various environmental and management factors or health conditions on equine social interactions.

Therefore, this video ethogram aims to establish standardised definitions of equine social behaviour complemented by videos to facilitate collaboration among researchers and cross-study comparisons to promote evidence-based optimisation of equine husbandry conditions and welfare.

## 2. Materials and Methods

A comprehensive ethogram was developed based on a recent systematic review of 27 papers [7,8,9,13,17,26,27,28,30,34,35,38,39,40,41,42,43,44,45,46,47,48,49,50,51,52,53,54] that investigated social behaviours among adult equines (≥2 years) interacting with conspecifics. After excluding maternal and sexual behaviours, the 37 different agonistic, affiliative, and neutral social behaviours, described in these 27 papers were compiled, and their definitions were harmonised to maximise the level of detail and reconcile any discrepancies. Employing a multi-step approach, each definition of each behaviour underwent a thorough review to identify key elements and characteristics. By conducting a meticulous word-by-word comparison of definitions from the various literature sources, shared elements and points of divergence between the different definitions were identified and analysed. Discrepancies and inconsistencies were resolved by considering the frequency of specific terms and maximum consensus among the definitions found in the literature sources. 

To enhance the clarity and detail of the compiled definitions and to establish a resource for standardizing behavioural terminology, each behaviour is illustrated with an accompanying video. To this end, six groups of 8–45 horses, aged 6 months to 32 years, were observed during paddock or pasture turn-out. Video recordings were captured using stationary GoPro (HERO4, 1280 × 960p 60fps) cameras affixed to the fence at a height of 1.5 m to 2.3 m, providing continuous recordings during turn-out. Additionally, iPhone 13 cameras were used for opportunistic recordings by observers, maintaining a sufficient distance to ensure horses’ undisturbed behaviour.

Some videos feature horses wearing halters or limb bandages equipped with sensors for concurrent studies, worn for an acclimatisation period of at least 10 days prior to video recordings without evident impact on their social behaviour. Videos featuring social interactions aligned with the ethogram definitions were identified through convenience sampling and confirmed by consensus among the authors.

## 3. Results

While each of the 27 papers presented variable subsets of behaviours, most (74%) ethograms included less than 15 of these 37 behaviours, and none included more than 22 of the entire set. Differences in terminology and definitions were observed among papers, with some using different terms for similar behaviours (e.g., ‘attack’ and ‘lunge’, ‘avoidance’ and ‘withdrawal’) [9,13,17,41,44,46,48,52] or interchangeably for separate behaviours (e.g., ‘retreat’ and ‘avoidance’, ‘agonistic approach’ and ‘chase’) [26,27,28,38,40,44]. 

**Figure 1:**
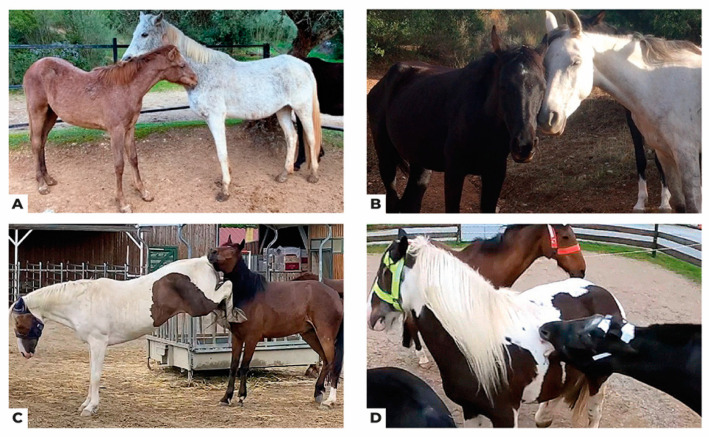
Still images extracted from the videos 7, 3, 33 and 23: (**A**) Mutual grooming/Allogrooming (Video S7), (**B**) affiliative body contact (Video S3), (**C**) Kick (Video S33), (**D**) Bite (Video S23). For behaviours marked by limited locomotion, such as allogrooming (**A**) and affiliative body contact (**B**), or those easily discernible without contextual cues, like a kick (**C**) or bite (**D**), static images are adequate to depict the essential elements of these behaviours. However, videos enable more detailed observations of facial expressions and body language, essential for understanding the precise mechanisms of social communication.

**Figure 2:**
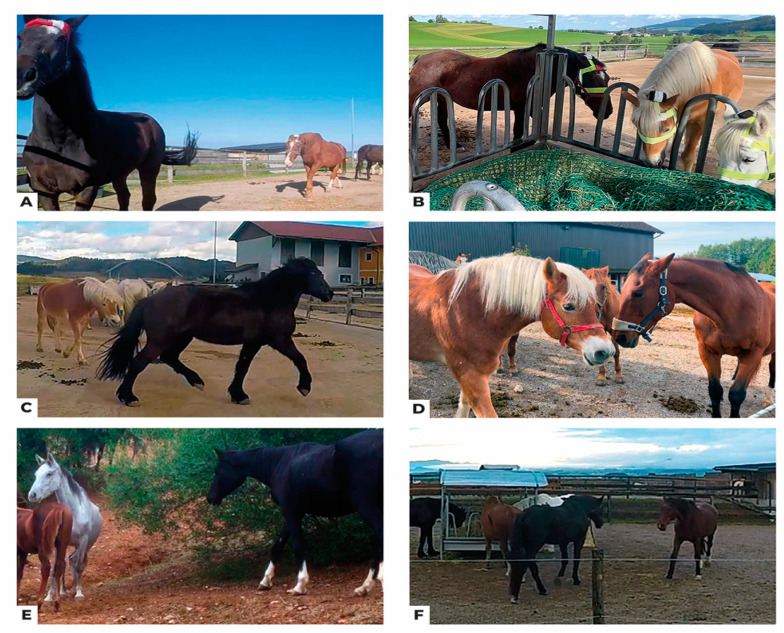
Still images extracted from the videos 16, 19, 41, 38, 1, and 6: (**A**) Approach eliciting retreat (Video S16), (**B**) Approach with supplantation (Video S19), (**C**) Retreat (Video S41), (**D**) Avoidance (Video S38), (**E**) Affiliative Approach (Video S1), (**F**) Mutual Approach (Video S6). Static images fall short of providing unequivocal identification for behaviours characterized by high levels of locomotion and/or those reliant on contextual cues. The dynamic and temporal aspects of social interactions are crucial for accurate characterization, necessitating the inclusion of dynamic or contextual representations to capture the entire essence of these behaviours.

Based on a synthesis of the diverse ethograms used in these studies and further refinement through detailed video observations, this video ethogram proposes standardised definitions for equine social behaviours accompanied by illustrative videos (Table 1). These definitions encompass contextual aspects (e.g., recognising circling as part of an aggressive or playful behavioural sequence) and account for communicative nuances (e.g., the consistent consideration of ear positioning). While static images may suffice for depicting some behaviours characterized by low levels of locomotion (e.g., allogrooming (Figure 1A) and affiliative body contact (Figure 1B)) or those readily identifiable without contextual cues (e.g., kick (Figure 1C), bite (Figure 1D)), for behaviours with intricate nuances, such as the diverse forms of approach (Figure 2A–F), incorporating video recordings becomes essential. Videos provide the invaluable dynamic context necessary for unequivocal identification and classification, ensuring the ethogram’s accuracy and replicability.

## 4. Discussion

Equine social behaviour studies employ a range of ethograms, each encompassing varying subsets of behaviours described with differing degrees of detail and clarity [44,54,75,76,77,78]. This heterogeneity impedes comparative analyses and restricts insights into the impact of husbandry and management practice on equine social behaviour. Moreover, the inconsistent use of terminology, with multiple terms employed to describe identical behaviours (e.g., ‘attack’ and ‘lunge’, ‘avoidance’ and ‘withdrawal’) [9,13,17,35,44,46,52] and terms describing distinct behaviours (e.g., ‘retreat’ [9,13,17,41,44,46,48,52] and ‘avoidance’ [9,13,17,42,45,47,49,53]), used interchangeably, introduces ambiguity and interpretational challenges. Furthermore, the traditional reliance on static visual representations, such as drawings and photos, in these ethograms has inherent limitations [30,38,44,75,76,77,78]. These images capture only a snapshot in time, failing to depict the fluidity and sequence of social interactions. This static approach risks misinterpretations and inconsistencies, as different observers may perceive the same behavioural sequence differently. Additionally, it can introduce subjective bias into the ethogram development process, as researchers may inadvertently tailor their depictions to fit their theoretical frameworks or hypotheses. 

In contrast, videos offer distinct advantages over static images when documenting social behaviours for ethograms. Videos capture the dynamic and temporal aspects of social interactions, enabling researchers to observe and analyse behavioural sequence, duration, and rhythm [77,79,80]. This temporal information is crucial for understanding the meaning and context of social interactions and the individual and group dynamics that shape equine societies [44]. Additionally, videos provide a more complete picture of behaviour, as they can capture subtle cues and interactions that might be missed in still images. Videos can be slowed down and analysed frame-by-frame, allowing more detailed observations of facial expressions, body language, and vocalisations. This level of detail is essential for understanding the precise mechanisms of social communication and the subtle cues horses use to navigate their social world, which is particularly important for studying rare or fleeting behaviours that might be difficult to capture in a single observation. Video-based ethograms, therefore, hold significant promise for facilitating more rigorous and comparative research.

The proposed refined equine social ethogram advocates standardised definitions of horses’ social behaviours, accompanied by video examples, to mitigate ambiguity and ensure consistency. This proposal aims to bridge communication gaps between different research groups and enable cross-study comparisons regarding the impact of husbandry practices and health conditions on equine social behaviour.

## 5. Conclusions

This ethogram introduces standardised definitions for equine social behaviours, complemented by videos as a foundation for future studies. Clear definitions are necessary to facilitate the comparison of data across studies and evidence-based optimisation of equine husbandry conditions and welfare.

## Figures and Tables

**Table 1:** Equine social behaviours ethogram, classified into 4 categories: (1) affiliative, (2) agonistic—aggressive, (3) agonistic—submissive, and (4) investigative and neutral behaviours. Each behaviour is defined, and a link to the corresponding video is provided.

Behaviour	Definition	Video Number
**Affiliative Social Behaviours**		
**Affiliative approach**	One horse moves toward another with ears oriented forward, closing the interindividual distance to two body lengths or less without triggering agonistic interactions. The approached horse holds its position without an immediate retreat, and both horses remain in close proximity for at least 10 s.	1
**Following**	One horse moves immediately behind another horse that has just initiated locomotion in the same direction. The follower’s ears are oriented forward.	2
**Affiliative body contact**	One horse, with its ears oriented forward or laterally, lightly touches another horse with its nose/lips.	3
**Grooming approach**	One horse advances toward another with ears oriented forward, closing the interindividual distance to engage in social grooming.	4
**Headrest**	One horse, with its ears oriented forward or laterally, rests its chin or entire head on the dorsal surface of the neck, withers, back, or croup of another horse.	5
**Mutual approach**	Two horses are advancing slowly toward each other with ears oriented forward, reducing the interindividual distance to two body lengths or less without triggering agonistic interactions.	6
**Mutual grooming/Allogrooming**	Two horses standing in close proximity, either head-to-tail or head-to-head, with their ears oriented forward or laterally, employ their teeth, lips, or tongue to engage in cleaning and maintenance activities on each other’s bodies.	7, 8
**Pairing/standing resting together**	Two or more horses are standing together in close proximity (<1 m) to each other, in a parallel or antiparallel position, without other overt social interaction. Ears are usually positioned laterally.	9, 10
**Pass under the neck**	One horse, with its ears oriented forward or laterally, passes its head and neck under another horse’s chin and neck.	11, 12
**Play**	Play behaviour in horses encompasses a wide range of recreational and non-aggressive interactions and activities such as running, bucking, jumping, and nipping. This behaviour is characterized by the horses having their ears oriented forward or laterally, lips protruded, teeth covered and lacking vocalization.	13
**Play fight**	An equine play fight is a social interaction between two horses mutually participating in playful, often exaggerated behaviours, like leaping, rearing, nipping, and energetic chasing, that mimic elements of real aggression but are performed in a non-threatening manner. Ears are positioned forward or laterally. In contrast to real fights, these actions are not intended to cause harm, and both horses willingly engage in the playful interaction.	14
**Rubbing**	One horse presses part of its body (head, forehead, chin, body) in a repetitive circular or up-and-down motion against another horse.	15
**Agonistic—Aggressive Social Behaviours**		
**Approach eliciting retreat**	One horse approaches another within a distance of 2 body lengths, ears pointed backwards. The approached horse then retreats to maintain or increase the interindividual distance.	16, 17
**Approach with supplantation**	A horse approaches another with its ears pointed backwards to assume its position. The approached horse retreats without urgency, maintaining or increasing the interindividual distance. The approaching horse does not chase the other after taking its position.	18, 19
**Arched neck threat**	A horse’s neck is tightly flexed with the muzzle drawn toward the chest, commonly observed in aggressive, investigative, or ritualized interactions.	20
**Attack/lunge**	A horse displays an aggressive charge towards another horse, with a characteristic forward surge, accompanied by a pronounced neck extension and backward-directed ears. This behaviour is often associated with biting or bite threats and might precede a chasing sequence.	21
**Backing**	A horse engages in retrogressive locomotion, moving backwards towards another horse with its ears pinned back.	22
**Bite**	One horse, with its ears pinned back, bodily contacts another horse by retracting its lips and closing its teeth on the other horse’s body. If the hold is sustained, this behaviour may be classified as a ‘grasping’ behaviour.	23
**Bite threat**	One horse performs biting-like movements towards another horse without making physical contact. This behaviour includes directed head movements, with the neck extended and ears pinned back.	24, 25
**Chase**	With its ears oriented backwards, one horse initiates a fast-paced pursuit of another horse, spanning at least three strides.	26
**Fight**	This behaviour between two horses is characterized by intense and prolonged aggression. It encompasses actions such as biting, striking, kicking, and chasing and may include vocalizations such as squeals.	28
**Head bowing**	One horse engages in repetitive, exaggerated, and rhythmical neck flexion, drawing its muzzle towards its chest while facing another horse. This behaviour may manifest in synchrony when two horses initially approach head-to-head. Its valence hinges on whether it is accompanied by squeals, stomping, and broader contextual cues.	29
**Head threat**	One horse exhibits a lowered head posture with ears pinned and a stretched or extended neck directed toward another horse.	30, 31
**Herding/driving**	A horse advances with its neck extended and ears oriented backwards, guiding the movement of one or more conspecifics. When the driving horse simultaneously executes lateral head movements, this behaviour is termed ‘snaking.	32
**Kick**	Rapid hindleg extension with contact. With its ears pinned back, one horse rapidly extends one or both hind legs backwards toward another, resulting in physical contact between the aggressor’s hooves and the other horse’s body.	33
**Kick threat**	Rapid hindleg extension without contact. One horse, with its ears pinned back, either rapidly extends one or both hind legs backwards toward another horse without making physical contact or raises one hind limb in preparation for a kick without extending the limb toward the other horse. This behaviour may also involve vigorous tail switching and squealing.	34, 35
**Push**	One horse presses a part of its body (head, neck, shoulder, body, or croup) against another horse to displace the target horse.	36
**Strike / Strike threat**	Rapid foreleg extension with or without contact: One horse rapidly extends one or both forelegs toward another horse without making physical contact. The striking horse has its ears oriented backwards.	37
**Agonistic—Submissive Social Behaviours**		
**Avoidance**	One horse moves to maintain or increase the distance from another non-threatening horse. The avoidant horse typically orients its ears backwards.	38
**Balk**	A horse abruptly halts or reverses direction with a rapid sweeping dorsolateral head and neck movement away from an apparent threat. The forelegs may simultaneously lift off the ground. The balking horse typically orients its ears backwards.	39
**Flight**	One horse immediately and rapidly moves to maintain or increase the distance from an attacking approaching horse. Both horses orient their ears backwards.	40
**Retreat**	One horse moves to maintain or increase the distance from a threatening approaching horse, either at a walk or trot. Both horses orient their ears backwards. A retreat can be differentiated from flight by the slower speed.	41, 42
**Snapping**	One horse exhibits a wide-open mouth with pulled-back corners, displaying teeth and gums while chewing. Its hind legs may be slightly bent in a cringing position. The head and neck are extended, and the ears are oriented back or laterally. This behaviour is typically exhibited by a younger or lower-ranked horse as appeasement to another horse.	43
**Behaviours with context-dependent variable valence**		
**Circling**	Two horses are moving in a circular motion around each other head-to-tail and attempting to nip or bite each other’s body parts. It can be a component of either a fight or a high-intensity play behaviour sequence. The valence is indicated by the orientation of the ears. Agonistic circling may be accompanied by bites, bite threats or squealing.	27
**Investigative and Neutral Social Behaviours**		
**Neutral approach**	One horse approaches another horse without any overt threat displays or ensuing agonistic or affiliative interactions.	44
**Nose-nose interaction**	A social encounter during which two horses closely approach each other, with their muzzles and noses in proximity, often involving touching or light interaction. The valence is dependent on the context and accompanying behaviours.	45
**Olfactory investigation**	One horse sniffs various parts of another horse’s body, such as the head, neck, flank, genitals, tail, or perineal region. The second horse may reciprocate this behaviour. The valence of this interaction varies depending on the context and can be discerned through accompanying vocalisations, stomping or ear position.	46

## Data Availability

All pertinent data is included in the manuscript and Supplementary Materials.

## Supplementary Materials

Video S1–S46 can be found at: https://zenodo.org/records/10952469 (accessed on 3 April 2024). Video S1: Affiliative Approach: Horse 1 approaches Horse 2, which does not move away. Both horses have their ears oriented forward and remain in close proximity after the approach. Video S2: Following: Horse 1 moves toward Horse 2, Horse 2 slowly walks forward and is immediately followed by Horse 1 (twice). Both horses have their ears oriented laterally. Video S3: Affiliative body contact: Horses 1 and 2 are standing close to each other. Both are resting with their ears positioned laterally. Horse 2 slowly gets his muzzle closer to Horse 1 and slightly touches its face. Video S4: Grooming Approach: Horse 1 slowly approaches Horse 2. The two horses remain in close proximity and start grooming each other. Video S5: Headrest: Horse 1, with its ears oriented laterally, puts its chin on the croup of Horse 2. Video S6: Mutual Approach: Horses 1 and 2 simultaneously approach each other at a slow pace. Horse 1 initially approaches with its ears forward, and when close to Horse 2, it positions its ears laterally. Horse 2 initially approaches with its ears laid back and, when close to Horse 1, positions its ears laterally. Video S7: Mutual grooming: Horses 1 and 2, standing in an antiparallel position with their ears oriented laterally, engage in mutual coat-nipping around the neck and shoulder area. Video S8: Mutual grooming: Horse 1, standing in an antiparallel position in close proximity to Horse 2 with its ears oriented laterally, is nipping Horse 2’s coat in the croup area. Video S9: Pairing/standing resting together: Horses 1, 2 and 3 are standing close to each other, resting, with their ears oriented laterally. Video S10: Pairing/standing resting together: Horses 1 and 2 are standing close to each other, head-to-tail, resting, with their ears oriented laterally. Video S11: Pass under the neck: Horse 1 approaches Horse 2 and passes its head and neck under Horse 2’s head. Both horses have their ears positioned laterally. Video S12: Pass under the neck: Horses 1 and 2 are allogrooming with their ears oriented laterally. Horse 2 passes under the mane of Horse 1. Video S13: Play: Horses 1 and 2 try to nip each other’s head and neck while pouncing. Both horses have their ears oriented laterally. Video S14: Play fight: Horses 1 and 2 mutually nip and strike at each other and rear using exaggerated movements without violent contact. Video S15: Rubbing: Horse 1, with its ears oriented laterally, rubs its head against Horse 2’s croup with an up-and-down motion. Video S16: Approach eliciting retreat: Horse 1 approaches Horse 2 with its ears pinned back and its neck extended. Horse 2 retreats rapidly to maintain/increase the interindividual distance. Video S17: Approach eliciting retreat: Horse 1 approaches Horse 2 with its ears pinned back and its head close to the ground. Horse 2 rapidly moves away with its ears pinned back. Video S18: Approach with supplantation: Horse 1 chases away three horses from the hay feeder with its ears pinned back and performs a bite threat before approaching Horse 2. Horse 2 immediately moves away while Horse 1 takes its place. Video S19: Approach with supplantation: Horse 1, with its ears pinned back, chases away Horses 2 and 3 from the hay feeder. Horses 2 and 3 immediately move away while Horse 1 takes their place. Video S20: Arched neck threat: Horse 1 tightly flexed its neck with its ears oriented laterally while facing Horse 2, which is separated from Horse 1 by a fence. Video S21: Attack/lunge: Horse 1 lunges rapidly towards Horse 2 with its ears pinned back and its neck extended in an apparent attempt to bite Horse 2. Horse 2 immediately and rapidly moves away with its ears pinned back. Video S22: Backing: Horse 1 approaches Horse 2 by walking backwards with its ears pinned back. Horse 2 moves away slowly, with its ears oriented backwards. Video S23: Bite: Horse 1, with its neck extended and ears pinned back, lunges towards Horse 2, retracting its lips and closing its teeth on Horse 2. Video S24: Bite threat: Horse 1, with its neck extended and ears pinned back, turns toward Horse 2, executing a biting motion without making physical contact. Horse 2 moves away with its ears pinned back. Video S25: Bite threat: Horse 1, with its neck extended and ears pinned back, turns toward Horse 2, executing a biting motion without making physical contact. Horse 2 moves away with its ears pinned back. Video S26: Chase: Horse 1, with its ears pinned back, is pursuing Horses 2 and 3 for a few seconds at a trot. Horses 2 and 3 also have their ears oriented backwards. This behaviour was observed shortly after Horses 2 and 3 were introduced to the group. Video S27: Circling: Horses 1 and 2 circle each other, head to tail, nipping each other’s hind legs. Video S28: Fight: Horse 1 approaches Horse 2 with its ears pinned back and executes a biting motion, initiating a fighting sequence where both horses try to kick each other. Video S29: Head bowing: Horse 1 faces Horse 2, head-to-head, separated by a fence and heads close to the ground. Horse 1 rapidly engages in a tight flexion of its neck with its ears oriented backwards and subtle repetitive and rhythmical flexions. Video S30: Head threat: Horse 1 turns its head toward Horse 2 and extends its neck and head in Horse 2’s direction with its ears pinned back. Video S31: Head threat: Horse 1 turns its head toward Horse 2 and extends its neck and head in Horse 2’s direction with its ears pinned back. Video S32: Herding: Horse 1, with an extended neck and ears pinned back, follows Horse 2 and tries to direct Horse 2’s movements while keeping it separated from the other conspecifics. Video S33: Kick: Horse 1, with its ears pinned back, rapidly extends both hindlegs backwards toward Horse 2, making physical contact. Video S34: Kick threat: Horse 1, with its ears pinned back, backs toward Horse 2 and rapidly extends its right hindleg toward Horse 2 without making physical contact. Video S35: Kick threat: Horse 1, with its ears pinned back, rapidly extends both hindlegs toward Horse 2 without making physical contact. Video S36: Push: Horse 1 positions himself on Horse 2’s side and pushes Horse 2 with its head and neck. Horse 2 is displaced to the side by this action. Video S37: Strike/ Strike threat: Horse 1, with its ears pinned back, rapidly strikes with both forelegs toward Horse 2. Horse 2 then also strikes with one foreleg and ears pinned back. There is no physical contact between the two horses. This video shows part of a play fight sequence. Video S38: Avoidance: Horse 1 approaches with its ears oriented to the front, and Horse 2, with its ears pinned back, moves away. Horse 1 orients its ears slightly backwards when Horse 2 has already initiated its movement. Video S39: Balk: Horse 1 abruptly switches direction when facing Horse 2, pivoting on its hindlegs. Video S40: Flight: Horse 2 immediately and rapidly moves away, with its ears laid back, from Horse 1, which is lunging with its ears laid back. Video S41: Retreat: Horse 1, with its ears pinned back, neck extended, and head held low, slowly approaches Horse 2. Horse 2, with its ears pinned back, moves away to maintain the interindividual distance. Video S42: Retreat: Horse 1, with its ears pinned back, neck extended, and head held low, slowly approaches Horse 2. Horse 2, with its ears pinned back, moves away to maintain the interindividual distance. Video S43: Snapping: Horse 1 gets closer to Horse 2, head-to-head, pulls back the corners of its mouth and exhibits a chewing motion. Horse 1’s head and neck are extended toward Horse 2, and its ears are oriented toward the front and laterally. Video S44: Neutral approach: Horse 1 approaches Horses 2 and 3 at the hay feeder with its ears oriented laterally. All horses stay close to each other and do not engage in direct interaction. Video S45: Nose-nose interaction: Horses 1 and 2 are in an antiparallel position, nose to nose with arched necks and Horse 1’s ears are oriented toward the front. Then, Horse 2 positions himself side-by-side with Horse 1, and both noses are touching. Their ears are oriented laterally while touching. Video S46: Olfactory investigation: Horses 1 and 2 sniff each other’s croup and back. Both horses have their ears oriented laterally.
